# The Surgery Queue

**Published:** 1922-09

**Authors:** R. W. Harris

**Affiliations:** (formerly Assistant Secretary, responsible for the administration of National Health Insurance Medical Benefits, Ministry of Health)


### THE SURGERY QUEUE.

**By R. W. HARRIS (formerly Assistant Secretary, responsible for the administration of National Health Insurance Medical Benefits, Ministry of Health).**

A HUMORIST has described a panel doctor's surgery as a place where people "surge." It will pass as a *jeu d'esprit*, but it will not serve as a general definition any more than will those occasional contributions to the public press serve as true pictures of the insurance medical service generally, which draw attention, sometimes in vivid language, to the scandal of queues outside surgeries.

I would therefore enter a plea that the doctors themselves—those immediately concerned, of course, but also their colleagues on the Panel Committees—should take this thing in hand, and see that it is cleared right away. For, where there are queues—or even overcrowding in the waiting-rooms, apart from very exceptional moments of extreme pressure during an epidemic of illness—the thing is a scandal. It is useless to say that insured persons have their own remedy by transferring to another doctor. They are, in the mass, apathetic ; they put up with things to a surprising extent. And meantime the good name of the service suffers, in the same way as from the other sweeping generalisations which are made about the "panel doctor." This overcrowding and overflowing of surgeries in certain districts has, however, been too often insisted upon for its truth to be questioned, even if one did not make the statement from personal observation. During the days of private practice before the Insurance Act, it may have failed to attract attention. To attribute the trouble to the Insurance Act is merely thoughtless, or worse. The Insurance Act did not create a new population or diminish the size of a doctor's surgery. But that is not to say that what would pass as good enough for private practice will do for a public service. If sick persons are unreasonably kept waiting, or are kept waiting in a queue—which is more than unreasonable—it is something which properly shocks the public conscience.

A tightening up of the certification machinery has provided that the doctor must not give a fresh certificate of incapacity to any patient except as the result of a fresh examination. There must, therefore, be no more sending for certificates, and the people who will be waiting in the crowded surgery, or in the queue, will usually be the patients themselves.

It was difficult during the war, and perhaps more difficult for some time afterwards, to get repairs and extensions carried out. But that difficulty has practically disappeared. And it therefore behoves every doctor who, from his experience in winters past, cannot satisfactorily answer his own question whether he is providing that "proper and sufficient surgery and waiting-room accommodation " which the terms of service require, to put the matter right, and to do it forthwith. It is equally the duty of every approved society representative on an Insurance Committee, when he is aware of a particular case of improper or insufficient accommodation, to see that the Insurance Committee at once take the matter up with the Panel Committee, whose co-operation can do much to ensure that the matter is put right. If the approved society representative feels that it is more picturesque to hold forth on a public platform about the iniquities of the " panel doctor," he will, I suppose, do it; but this is not the effective way of bringing about an improvement.

Dr. Gordon Ward, in a letter which appears in this issue, says, in his anger against the approved societies, that they continually emphasise the few cases in which medical men have gone wrong, and " do their best to discredit the profession in the eyes of the people." He proceeds to imply that it is to the action of the approved societies that is due the " significant and noteworthy symptom that the medical men of the East End of London are already very largely foreigners, Hindus, Parsees and Negroes." And adds: " It has been calculated that 25 per cent. of the names on the London panel list are those of foreigners." It would be of interest to know whether such a statement as this is calculated to do credit to, or to discredit, the medical profession, and also whether it is seriously intended to convey that it is the conditions of service under the Insurance Acts, and not the general conditions and environment of some East End practices, that have determined the personnel of the profession in that part of London. I wonder what some of the medical men themselves think of it?

Amongst some new regulations which will come into force on September 1 appears the following addition to the insurance doctor's terms of service:—

" A practitioner shall when required satisfy the Committee, or on appeal the Minister, that the arrangements for the conduct of his practice, and in particular for the visiting of those of his patients whose condition so requires, are such as to enable the practitioner's obligations under these terms of service to be adequately carried out."

This is understood to be primarily directed against the " lock-up surgery"—a quite different problem from that of the overcrowded surgery; but the regulation is very wide in its terms, and would clearly include a requirement that a doctor should satisfy the Insurance Committee, if called upon to do so, that that part of his obligation which involves the provision of proper surgery and waiting-room accommodation is " adequately carried out." If in individual cases there are difficulties, does not Lord Dawson's persistent advocacy of " team-work " offer a promising solution?

